# Amyloid precursor protein, lipofuscin accumulation and expression of autophagy markers in aged bovine brain

**DOI:** 10.1186/s12917-017-1028-1

**Published:** 2017-04-13

**Authors:** D. De Biase, A. Costagliola, T.B. Pagano, G. Piegari, S. Wojcik, J. Dziewiątkowski, E. Grieco, G. Mattace Raso, V. Russo, S. Papparella, O. Paciello

**Affiliations:** 1 Department of Veterinary Medicine and Animal Production, University of Naples Federico II via Delpino, 1, 80137 Naples, Italy; 2 Department of Anatomy and Neurobiology, Medical University of Gdansk, Debinki 1 80-11, Gdansk, Poland; 3 Azienda Sanitaria Locale, Salerno, Italy; 4 Department of Pharmacy, University of Naples Federico II, Via Montesano 49, 80131 Naples, Italy

**Keywords:** Autophagy, Brain, Neuropathology, Ageing, Bovine

## Abstract

**Background:**

Autophagy is a highly regulated process involving the bulk degradation of cytoplasmic macromolecules and organelles in mammalian cells via the lysosomal system. Dysregulation of autophagy is implicated in the pathogenesis of many neurodegenerative diseases and integrity of the autophagosomal - lysosomal network appears to be critical in the progression of aging. Our aim was to survey the expression of autophagy markers and Amyloid precursor protein (APP) in aged bovine brains. For our study, we collected samples from the brain of old (aged 11–20 years) and young (aged 1–5 years) Podolic dairy cows. Formalin-fixed and paraffin embedded sections were stained with routine and special staining techniques. Primary antibodies for APP and autophagy markers such as Beclin-1 and LC3 were used to perform immunofluorescence and Western blot analysis.

**Results:**

Histologically, the most consistent morphological finding was the age-related accumulation of intraneuronal lipofuscin. Furthermore, in aged bovine brains, immunofluorescence detected a strongly positive immunoreaction to APP and LC3. Beclin-1 immunoreaction was weak or absent. In young controls, the immunoreaction for Beclin-1 and LC3 was mild while the immunoreaction for APP was absent. Western blot analysis confirmed an increased APP expression and LC3-II/LC3-I ratio and a decreased expression of Beclin-1 in aged cows.

**Conclusions:**

These data suggest that, in aged bovine, autophagy is significantly impaired if compared to young animals and they confirm that intraneuronal APP deposition increases with age.

## Background

Autophagy is a self-degradative, highly regulated process that involves the non-specific degradation of cytoplasmic macromolecules and organelles via the lysosomal system. There are three different autophagic pathways based on the mechanisms for delivery of cargo to lysosomes: macroautophagy, microautophagy and chaperone-mediated autophagy (CMA) [1]. Macroautophagy (herein referred to as autophagy) is the major lysosomal pathway for the turnover of cytoplasmic components. Specifically, this process consists of the following steps: induction or initiation and cargo selection, vesicle nucleation and expansion, lysosome targeting, lysosome docking and autophagosome-lysosome fusion, vesicle breakdown and recycling. During Nucleation, the activity of specific autophagy effectors including Beclin-1 (Atg6 orthologue) and LC3 (microtubule-associated protein 1 light chain 3) generates an active phagophore (or isolation membrane) [2]. The nascent membrane wraps around a portion of cytoplasm (including the soluble proteins, aggregates, or organelle) to eventually form a double membrane-bounded structure called “autophagosome”. The nascent autophagosome subsequently fuse with lysosomes to form an autophagolysosome in which the cytoplasmic cargo is degraded by lysosome hydrolases; degradation products are recycled for the synthesis of new molecules [2]. Emerging evidence indicates that autophagy protects cells by removing long-lived proteins, aggregated protein complexes, and excess or damaged organelles [3]. Defects in autophagy, therefore, are associated to various pathological conditions within organisms, including tumorigenesis, defects in developmental programs and the build-up of toxic, protein aggregates involved in neurodegeneration [3] such as Amyloid precursor protein (APP). It has been recently suggested that the progressive age-related decline of autophagic and lysosomal activity may also be responsible for the continuous intraneuronal accumulation of lipofuscin, or “age pigment” [4]. For this study, we aimed to investigate the expression of autophagic markers such as Beclin 1 and LC3 and the accumulation of pathologic proteins such as APP and lipofuscin in aged bovine brains.

## Results

### Histopathology

**Fig. 1:**
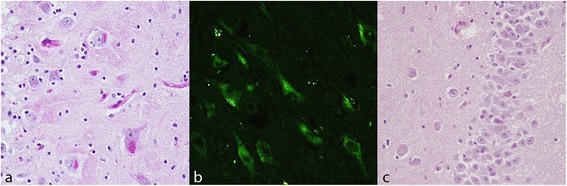
**a** Lipofuscin storage, hippocampus, dentate gyrus. Pyramidal neurons showing an abundant granular, PAS positive, intracytoplasmatic storage material. Periodic acid-Schiff (PAS) stain, 40X. **b** Intraneuronal storage material exhibits green-yellow autofluorescence by fluorescence microscopy. Fluorescence microscope; FITC filter (excitation, 455–500 nm; emission, 500–570 nm), 40X. **c** Neurons of young controls does not exhibit PAS positive and intracytoplasmatic storage material. Periodic acid-Schiff (PAS) stain, 40X

In aged bovine, microscopic evaluation revealed a moderate to severe gliosis and an increase of the number of glial cells around neurons (satellitosis) in the gray matter. Moreover, we observed scattered, strongly eosinophilic and shrunken neurons with nuclear pyknosis of a centrally placed nucleus. This findings were not observed in young animals. The striking and constant finding in all aged brains (group A) was the accumulation of small perinuclear and granular yellow-brown, Periodic acid – Schiff (PAS) stain positive (Fig. 1a) and autofluorescent deposits (Fig. 1b) consisting of lipofuscin. The amount of lipofuscin was moderate to severe, with a wide distribution in both cerebral cortex and hippocampus regions. Lipofuscin accumulation was found within the perikarya and sometimes the proximal dendritic tree of neurons and within the cytoplasm of astrocytes. No evidence of lipofuscin accumulation was observed in the neurons of young animals (Fig. 1c).Lipofuscin accumulation was significantly more noticeable among the older animals (group A) (*p* < 0.001). There was a statistically significant positive association between age and increase in severity of lipofuscin accumulation (G = 0.7980; *p* < 0.001) and this relation was strong (d Somer’s statistics =0.6611; *p* < 0.001).

### Immunofluorescence

**Fig. 2:**
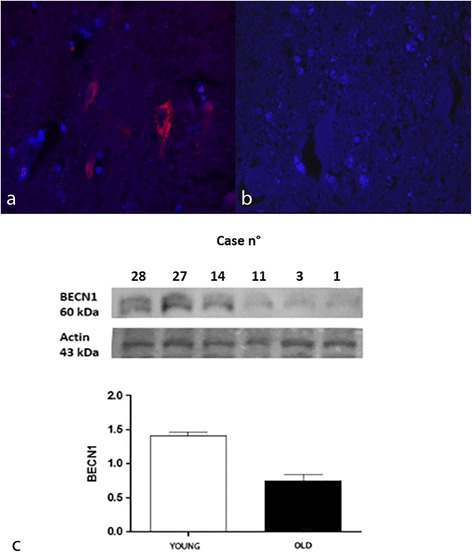
Beclin 1 expression, hippocampus, dentate gyrus. **a** Immunostaining inyoung and **b** aged brain. TRITC filter (excitation, 543 nm; emission 560 nm). DAPI counterstain, 40X. **c** Protein expression in young and aged bovine brains. Densitometric values shows that BECN1 is expressed in higher amount in young controls when compared to aged brain samples (*P* < 0,001 vs controls). Bars refers to mean values. Actin protein levels confirm the amount of protein loading in each lane

In all samples, autophagy induction was evaluated by Beclin-1 immunofluorescence and showed a progressive age-related decrease of intraneuronal Beclin-1 expression. In young animals (Group B), the percentage of Beclin-1 immuno-positive neurons and astrocytes as well as Beclin-1 staining intensity was of mild degree and widespread. Beclin-1 staining intensity as well as percentage of Beclin-1 immuno-positive cells were significantly more noticeable among the young animals (*p* < 0.01and *p* < 0.001, respectively). On the contrary, in 76% (*n* = 14) of samples from aged cows (group A) no Beclin-1 positivity was detected (Fig. 2a, b). In remaining 24% of cases percentage of Beclin-1 immuno-positive cells as well as Beclin-1 staining intensity was of mild degree. A Beclin-1 positivity was mainly observed in neurons in the hippocampus. There was a statistically significant negative association between age and increase of percentage of Beclin-1 immunoreactive cells (G = −0.9521; *p* < 0.001) and this relation was strong (d Somer’s statistics = −0.6653; *p* < 0.001). There was also a statistically significant negative association between age and increase of staining intensity of Beclin-1 immunoreactivity (G = −0.8523; *p* < 0.001) and this relation was strong (d Somer’s statistics = −0.6276; *p* < 0.001).

**Fig. 3:**
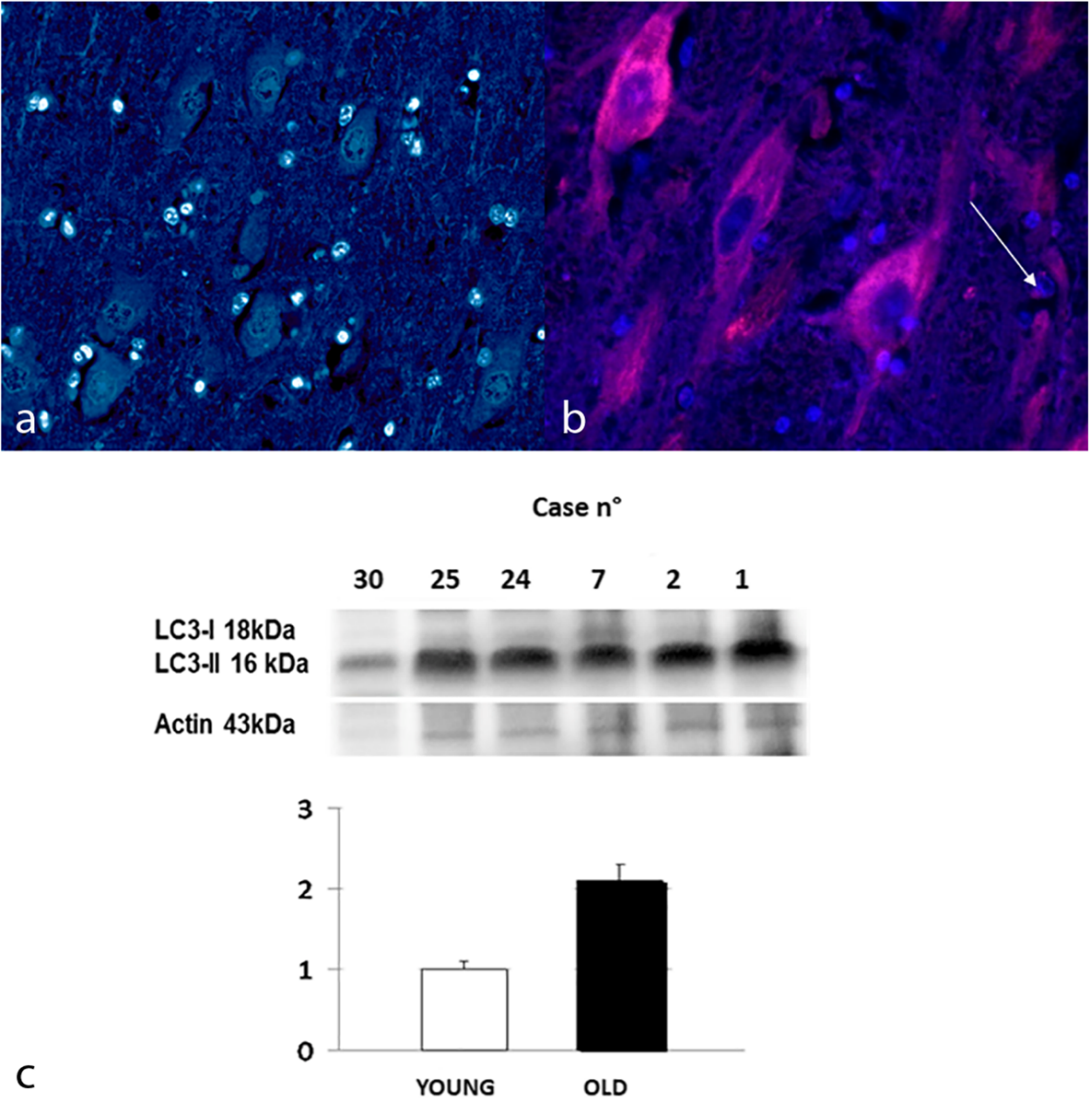
LC3 expression, hippocampus, dentate gyrus. **a** Immunostaining in young and **b** aged brain. Immunoreaction for LC3 was detected also in astrocytes (arrow).TRITC filter (excitation, 543 nm; emission 560 nm). DAPI counterstain, 40X. **c** Protein expression in young and aged bovine brains. Densitometric values shows that LC3-II/LC3-I ratio is significantly increased in the aged animals compared to the young controls (*P* < 0,05 vs control). Bars refers to mean values. Actin protein levels confirm the amount of protein loading in each lane

Moreover, strongly LC3 immuno-positive neurons and astrocytes were observed in aged animals (Fig. 3a), indicating possibly an excessive accumulation of autophagosomes. In younger animals, LC3 staining intensity was mostly absent (Fig 3b), even though a low percentage of LC3 widespread immune-positive neurons was rarely observed. These parameters were significantly more noticeable among the aged animals (*p* < 0.01and *p* < 0.001, respectively). There was a statistically significant positive association between age and increase of these parameters (G = 1; *p* < 0.001) and this relation was moderate (d Somer’s statistics =0.5230; *p* < 0.001).

**Fig. 4:**
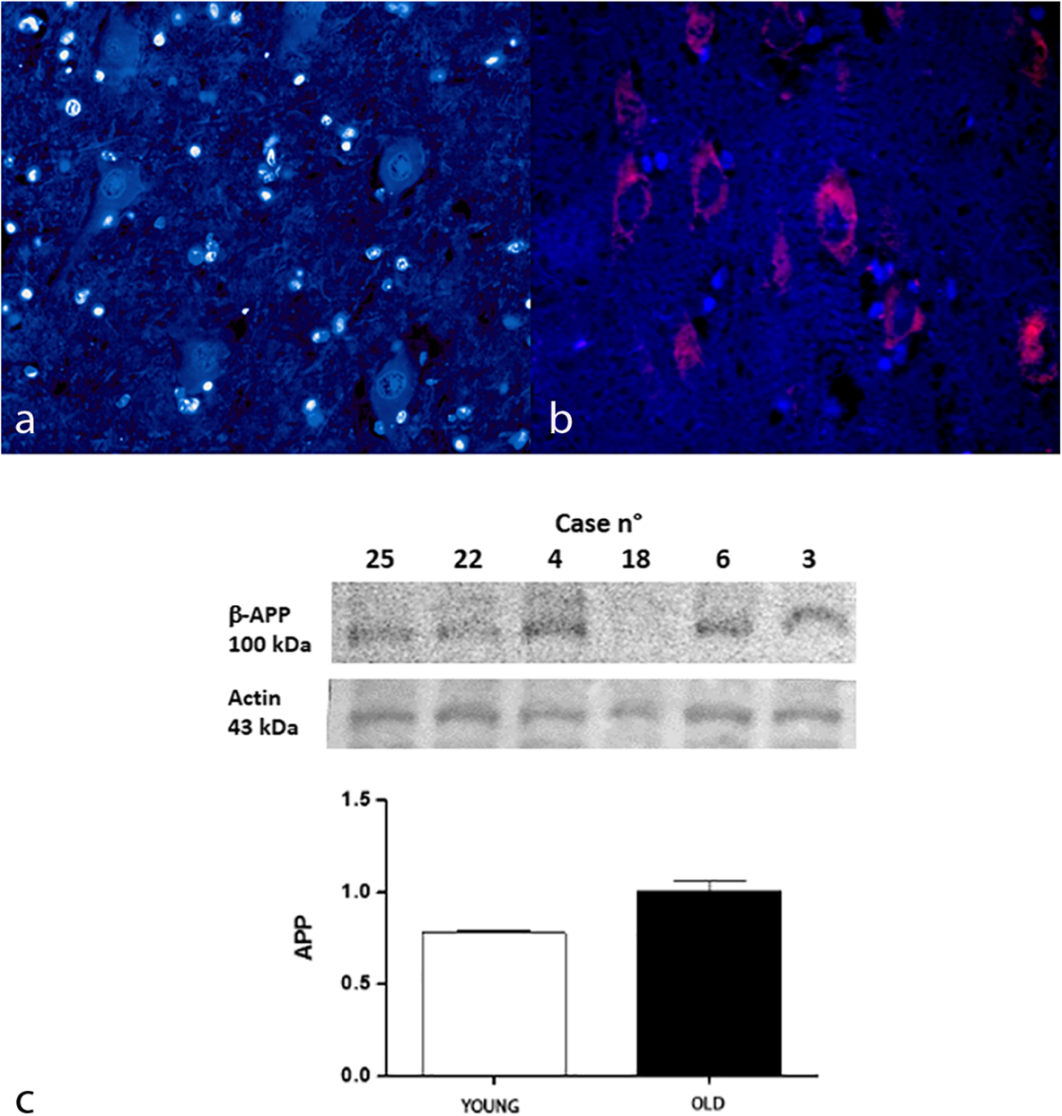
APP expression, hippocampus, dentate gyrus. **a** Immunostaining in young and **b** aged brain. TRITC filter (excitation, 543 nm; emission 560 nm). DAPI counterstain, 40X. **c** Protein expression in young and aged bovine brains. Densitometric values shows that APP is expressed in higher amount in aged brains when compared to young controls (*P* < 0,05 vs controls). Bars refers to mean values. Actin protein levels confirm the amount of protein loading in each lane

Amyloid precursor protein (APP) positive immunoreaction was detected within the cytoplasm of neurons and astrocytes in the hippocampus and cerebral frontal cortex of all the aged animals (group A) (Fig. 4a), but never in young animals (Fig 4b). There was high degree of staining intensity of APP in all the cases of aged animals. Percentage of APP immuno-positive cells as well as their staining intensity were significantly more noticeable among the older animals (*p* < 0.001). There was a statistically significant positive association between age and increase in severity of APP positive immunoreaction (G = 1; *p* < 0.001) and this relation was moderate (d Somer’s statistics =0.5230; *p* < 0.001).

### Western blot analysis

We evaluated the expression levels of APP, Beclin-1, and LC3 in elder cows showing immunohistochemical positivity for APP (represented as 100 kDa band), Beclin-1(represented as 60 kDa band) and two isoforms of LC3 (represented as 18 kDa band for LC3-I and 16 kDa band for LC3-II) mainly to confirm the dysregulation of autophagy in brain from elder cows compared to young animals. Our results, normalized for actin, showed that Beclin-1 (60 kDa) was significantly reduced in the aged animals compared with the young animals (*** *P* < 0,001 vs control) (Fig 2c) and an increased LC3-II/LC3-I ratio (*P* < 0,05 vs control) (Fig. 3c). Moreover, a significant increase in the oldest animals compared to young animals (*P* < 0,05 vs control) (Fig. 4c) for APP was detected with a band approximatively to 100 kDa as previously described [5].

## Discussion

Microscopic findings in the brains of our aged bovine are similar to those previously described in old animals of other species as well as in cattle [6–9]. Satellitosis is generally the result of an altered cellular homeostasis leading to neuronal damage and depletion [10]. Specifically, satellitosis represents a response to primary neuronal degeneration that increases in aged animals and often portends to the imminent progression to neuronophagia, a process whereby the degenerated fragments of a necrotic neuron are removed piecemeal by macrophages [11].

In this study, the age-dependent intraneuronal accumulation of lipofuscin is one of the most striking features of aged brains. This finding is not actually new, as it has been described for more than 150 years [12]. In the past, lipofuscin was generally thought to be an innocent end product of oxidation which has no significant influence on cellular activities [13], but in the last decade several authors have investigated about the possible detrimental and pathogenic potential of this material.

Turman and Banks, in a series of elegant experiments [12, 14, 15], hypothesized the so-called “mitochondrial–lysosomal axis theory of aging” that tries to explain the possible relationship between lipofuscin accumulation, decreased autophagy, increased Reactive Oxygen Species (ROS) production, and mitochondrial damage in senescent long-lived postmitotic cells. According to this theory, in senescent cells lysosomal enzymes are directed towards the plentiful lipofuscin-rich lysosomes and, subsequently, they are lost for effective autophagic degradation because lipofuscin remains non-degradable. The consequences are a progressive impairment of autophagy and the gradual accumulation of damaged mitochondria, other organelles and misfolded proteins that lead to neurodegeneration.

Unfortunately, our results cannot support a direct association between lipofuscin accumulation and autophagy impairment in aged bovine brains. According to recent scientific literature, we can only hypothesize that progressive and severe lipofuscin accumulation may irreversibly lead to functional decline and death of neurons by diminishing lysosomal degradative capacity and by preventing lysosomal enzymes from targeting to functional autophagosomes. Cytotoxic activity of lipofuscin consists also in its ability to catalyze the Fenton reaction: in presence of Fe2+, H2O2 is decomposed forming hydroxyl radicals (OH) [15]. In vitro, this material is able to generate the formation of free radicals and initiates apoptotic cell death [16]. However, the contribution of lipofuscin in the initiation of apoptotic cell death in vivo is still poorly understood.

Further studies are indeed necessary to better understand how lipofuscin accumulation can influence the neuronal autophagic and apoptotic pathways in bovine brains. It would be interesting to perform double-staining techniquesin order to show whether lipofuscin is directly related to autophagic and apoptosis markersand/or to pathologic proteins deposition. Unfortunately, to our knowledge, a specific antibody for lipofuscin is not available since this complex substance is mainly composed of cross-linked protein and lipid residues. Alternatively, combined histochemical and immunohistochemical staining protocols can be performed to simultaneously localize lipofuscin and the antigen of interest [17]. However, since lipofuscin progressively accumulates throughout the life of neurons, this combined immunohistochemical/histochemical protocol is not perfectly indicated to investigate the mechanism and relative timing of intraneuronal lipofuscin accumulation and the deposition of other proteins. Primary cultured neuronal cells exhibit, in vitro, a variety of features that are frequently observed in physiologically aged neurons in vivo*,* including lipofuscin accumulation [18]. Thus, long-term aging culture of primary cultured neurons would be a remarkable model to unravel, at least in part, the molecular mechanisms behind lipofuscin accumulation and its pathological effects on neuronal cells.

Despite its important limitations, to our knowledge this is the first study that describe the expression of autophagy markers in aged bovine brains. Our results suggest that in aged cow autophagy is significantly impaired if compared to young animals and they confirm that intraneuronal APP deposition increases with age. Beclin-1 and LC3 play a pivotal role in the autophagy process and their expression by immunoblotting, immunohistochemistry or immunofluorescence has become a reliable method for monitoring autophagy and autophagy-related processes [19]. Beclin-1 is a positive regulator of the autophagy pathway and promotes its induction whereas LC3 facilitates autophagosome elongation and closure [19]. During autophagic activity, the cytosolic form of LC3 (LC3-I) is conjugated to phosphatidylethanolamine to form LC3-phosphatidylethanolamine conjugate (LC3-II), which localizes to both the outside and the inside membranes of autophagosomes [20]. Autophagosomes fuse with lysosomes to form autolysosomes and intra-autophagosomal components as well as LC3-II are degraded by lysosomal hydrolases. Results from immunofluorescence and western blot analysis showed a progressive age-related decrease of intraneuronal Beclin-1 expression that most likely indicate a decrease in autophagy initiation. The increase of LC3 antibody expression detected by immunofluorescence may indicate an excessive accumulation of autophagosomes. Interestingly, western blot analysis showed an increased LC3-II expression that most likely indicate an impaired autophagosomal degradation resulting in the persistence of autophagic vacuoles that could interfere with intracellular trafficking promoting the presence of cytotoxic products [20].

Recently, Vallino Costassa et al. [21] characterized the nature of Amyloid β (Aβ) deposits in aged bovine brains pointing out that they are similar to those in humans in early stages of aging. Consistent with these findings, we observed an age-dependent, intraneuronal accumulation of APP immuno-positive material. Furthermore, western blot analysis showed an increased level of APP in aged animal compared to young animals. Several authors suggested that autophagy dysregulation may alter APP metabolism and fail to clear aggregated Aβ via autophagy - lysosome system promoting the accumulation of misfolded proteins and subsequent neurodegeneration. [22, 23]. Moreover, we recently observed accumulation of APP in the sarcoplasm of some of the aged cows used for this study [24]. The accumulation within abnormal muscle fibers of several pathologic and Alzheimer-related proteins such as beta-amyloid precursor protein (beta-APP), phosphorylated tau, alpha-1-antichymotrypsin, apolipoprotein E and presenilin-1 is an unusual feature of sporadic inclusion-body myositis (sIBM) [25]. We can speculate that same pathophysiological mechanism leading to the accumulation of APP can occur in the brain as well as in the muscle of old cows.

However, molecular mechanisms underlying the progressive accumulation of toxic proteins during aging remain still elusive and unclear. In healthy individuals, APP is transcribed in the endoplasmic reticulum, modified by Golgi network and then shuttled to the cell surface through the secretory pathway [26]. APP can then either be degraded through the autophagy-lysosome system, or recycled by endosomes entering the cycle again [26]. An interesting study of Pickford et al. contributed to clarify the association between autophagy impairment and neurodegeneration providing evidence that an essential component of the autophagy pathway, such as Beclin-1, is reduced in early Alzheimer disease promoting amyloid β accumulation in mice [27, 28]. Induction of autophagy and autophagosomal degradation seem to be impaired in Beclin-1 deficient cells. As a consequence, APP containing vesicles (endosomes, autophagosomes, and others) build up inside the cell. APP is increasingly cleaved by secretases generating Aβ that is possibly released from the cell [26]. Furthermore, the progressive accumulation of autophagosomes due to an impairment of autophagosomal degradation can serve as sites of Aβ generation, promoting the inhibition of APP turnover and degradation [26].

## Conclusions

Nowadays, it’s a firm belief that impairment and dysregulation of autophagy pathways is related to many neurological diseases both in humans and animals. To our knowledge, this is the first report concerning about the expression of autophagy markers Beclin 1 and LC3 in aged bovine brains. Our data show that autophagy is impaired in aged bovine brains and that intraneuronal accumulation of APP increases with age. Future studies, however, will allow to further understand the cellular and molecular events regulating the autophagy machinery.

## Methods

### Animals

For the present study, samples were collected from thirty Podolic dairy cattle (1–20 years old) (Table 1) in an abattoir in Campania Region, Italy, during post mortem inspection. Permission to obtain the samples was granted from the owner of the abattoir and from the veterinary inspector responsible for the sanitary surveillance. Each animal underwent a physical examination that did not report any apparent clinical illness or neurological sign (gait abnormalities, weakness and decreased mental status). Afterward, the animals were slaughtered in strictly accordance with European slaughter regulations (CE n° 1099/2009). Moreover, the absence of prion diseases was confirmed in all animals by performing the rapid test recommended by European law. Animals were divided in two groups: group A (aged) comprised bovine aged more than ten years (*n* = 19) and group B (young) comprised bovine aged up to five years (*n* = 11).

**Table 1:** Age (in years) of the aged (Nos. 1–19) and young (Nos. 20–30) animals employed in this study

**Bovine n°**	**Age (years)**
1	20
2	16
3	16
4	13
5	13
6	13
7	13
8	13
9	13
10	13
11	13
12	12
13	12
14	12
15	12
16	11
17	11
18	11
19	11
*20*	*5*
*21*	*5*
*22*	*5*
*23*	*4*
*24*	*3*
*25*	*2*
*26*	*3*
*27*	*2*
*28*	*1*
*29*	*1*
*30*	*1*

### Morphological analysis

At slaughterhouse, the brain was immediately removed and divided into two parts by a sagittal paramedian cut. The small part was frozen at −80 °C until further processing while the other part was fixed for 15 days in 10% neutral buffered formalin for histological and immunohistochemical examination. Transversal sections were taken from superior frontal gyrus and hippocampus (dentate gyrus). Precisely, for hippocampal formation we analysed pyramidal cells layer, Cornu ammonis (CA2, CA3 and CA4) fields. Sections were subsequently embedded in paraffin, sectioned at 4 μm and stained with haematoxylin and eosin (HE) and Periodic acid–Schiff (PAS). Histological specimens were examined and photographed with a light microscope (Nikon eclipse E600) associated to a microphotography system (Nikon digital camera DMX1200). Unstained sections from all cases were also evaluated with a fluorescence microscope (AxioSkop2 MOT, Zeiss) associated to a microphotography system (AxioCam MRc5, Zeiss) using blue light excitation (FITC filter; excitation, 455–500 nm; emission, 500–570 nm) in order to detect lipofuscin autofluorescence. The degree of lipofuscin accumulation was estimated as the quantity of neurons containing PAS positive storage material and scored as follows: absent, mild (less than one-third), moderate (between one-third and two-thirds), and high degree (more than two-thirds).

### Immunofluorescence

For all immunofluorescence experiments, 4-μm-thick sections of frontal cortex and hippocampus were mounted on positively charged glass slides (Bio-Optica, Milan). Antigen retrieval pretreatments were performed using a Heat Induced Epitope Retrieval (HIER) citrate buffer pH 6.0 (Bio-Optica, Milan, Italy) for 20 min at 98 °C. Primary antibodies used in this study included rabbit polyclonal Beclin-1 (BECN 1 H300: sc-11,427, Santa Cruz Biotechnology, Dallas, Texas, US), rabbit polyclonal LC3 (ab51520, Abcam, Cambridge, UK), mouse monoclonal APP (6E10) (SIG-39320, BioLegend, San Diego, California, US). The method was previously described [29]. Briefly, the primary antibody diluted in Phosphate Buffer Saline (PBS) was applied overnight at 4 °C. Slides were washed two times, five minutes each, in PBS and incubated for two hours at room temperature with a Goat anti-Rabbit IgG (H + L) Secondary Antibody (Alexa Fluor® 546 conjugate, A-11035, Thermo Fisher PO Box 117 Rockford, IL, USA) for Beclin 1, LC3, β-amyloid_1–42_ and Caspase 3 and a Goat anti-Mouse IgG (H + L) Secondary Antibody (Alexa Fluor® 546 conjugate, A11030, Thermo Fisher PO Box 117 Rockford, IL, USA) for APP. Both secondary antibodies were diluted 1:50 in PBS.

Slides were rinsed three times with PBS and coverslipped using Vectashield mounting medium containing 4′,6-diamidino-2-phenylindole (DAPI) (H-1200 Vector Laboratories, Inc., 30 Ingold Road, Burlingame, CA, USA).

For scanning and photography, a laser scanning microscope (LSM 510; Zeiss, Göttingen, Germany) was used. Antibodies bound to Tetramethylrhodamine (TRITC) were illuminated at 543 nm and then read with a 560 nm long pass filter. DAPI was illuminated at 360 nm and then read with a 460 nm long pass filter.

The different frames were scanned separately, with appropriate installation of the optical path for excitation and emission of each scan according to the manufacturer’s instructions.

To test the specificity of staining, two negative controls were simultaneously performed incubating one section with PBS (0.01 M PBS, pH 7, 2) omitting the primary antibody and the other with an irrelevant and unspecific IgG. For immunofluorescence analysis, the intensity grade of immunoreactivity was measured as the intensity of staining and percentage of positively stained cells referred to the entire studied sections and subsequently assessed as it follows: negative (0), mild (1), moderate (2) and high (3). The degree of lipofuscin accumulation, the intensity grade of staining and the percentage of positively stained cells was scored by two independent observers (OP, SP) in each specimen, for each antibody and under blinded conditions as performed in a previous study [19].

### Western blot analysis

Samples of brain from elder cows and controls were cut at the cryostat at 20 μm and then lysed at 4 °C in 200 μL of TBS lysis buffer (Tris Buffer Saline, 20 mM Tris-HCl pH 7.6, 140 mM NaCl, 30 mM sodium pyrophosphate, 5 mM EDTA, 0.55% Nonident P40, 1% Triton X-100, 50 mM NaF, 0.1 mM Na3VO4, 1 mM PMSF, 1 mM benzamidine, 1 mM iodoacetamide, 1 mMphenantroline). Protein concentration in the supernatant was determined by Bicinchoninic Acid assay (BCA) protein assay (BCA: Pierce Biotechnology, Rockford, Illinois, US), and lysates were adjusted to equivalent concentrations with lysis buffer. Aliquots of 10 mg of total brain lysate were then separated on Sodium Dodecyl Sulphate - PolyAcrylamide Gel Electrophoresis (SDS-PAGE). Proteins were transferred to Sodium Dodecyl Sulphate - PolyAcrylamide Gel Electrophoresis (PVDF) membranes that were blocked overnight at 4 °C with 5% non-fat dried skimmed milk in TTBS (TBS with 0.05% Tween 20). Incubation with primary specific antibodies against Beclin-1 (1: 500 dilution), LC3 (both isoforms, dilution 1: 3000) and mouse monoclonal APP (6E10) (dilution 1:2000) and horseradish peroxidase-conjugated secondary antibodies was performed in blocking solution for 1 h at room temperature. Immunoreactive bands were visualized by SuperSignal West Pico Chemiluminescent substrate kit (Pierce Biotechnology, Rockford, Illinois, US). The same blots were stripped and re-probed using anti-beta actin monoclonal antibody to confirm equal loading of proteins in all lanes. Bands intensities were quantified on scanned images using Image J software (National Institute of Health, USA) to determine average pixel intensity.

### Statistical analysis

Data obtained from Western blots were analysed with Statview software (Abacus Concepts) by t-Student test. Blots were revealed by Enhanced chemiluminescence (ECL) and autoradiography using beta-actin as a loading control. The autoradiographs shown are representative of four independent experiments. Bars represent the mean ± SD of four independent experiments. The chi-squared test was used to assess differences between the studied groups of cows. We also performed Gamma statistic G [30] in order to investigate for possible associations between immunohistochemical findings and age. Subsequently, d-Somer’s statistics was performed to establish the relation of present association. *P*-values of <0.05 were considered statistically significant. Gamma statistic G (Goodman and Kruskal’s gamma) measures the strength of association of the contingency table values when the variables are on an ordinal scale. Somers’ D is closely related to the gamma statistic and it’s one of the most commonly used measures of association for doubly ordered contingency tables.

## Abbreviations

APP: Amyloid Precursor Protein
Aβ: Amyloid beta
BCA: Bicinchoninic Acid assay
CA (2, 3 and 4): Cornu Ammonis area
CMA: Chaperone-mediated Autophagy
DAPI: 4′,6-diamidino-2-phenylindole
ECL: Enhanced ChemiLuminescence
EDTA: Ethylene-Diamine-Tetraacetic Acid
HE: Haematoxylin and Eosin
HIER: Heat Induced Epitope Retrieval
LC3: Microtubule-associated protein 1 light chain 3
OH: Hydroxyl radicals
PAS: Periodic acid – Schiff
PBS: Phosphate-buffered Saline
PVDF: PolyVinylidene DiFluoride
ROS: Reactive oxygen species
SDS-PAGE: Sodium Dodecyl Sulphate - PolyAcrylamide Gel Electrophoresis
sIBM: Sporadic Inclusion Body Miositis
TBS: Tris-buffered saline
TRITC: Tetramethylrhodamine
TTBS: Tween Tris-Buffered Saline
